# Identification of Cancer Related Risk and Protective Factors for American Indian Youth: A Mixed Studies Review

**DOI:** 10.3389/fpubh.2022.828776

**Published:** 2022-04-25

**Authors:** Melanie Nadeau, Kathryn Wise, Vianca Farfan Cuela, Devon Olson, Karan Saravana

**Affiliations:** Department of Indigenous Health, School of Medicine and Health Sciences, University of North Dakota, Grand Forks, ND, United States

**Keywords:** risk factor, protective factor, Native American, American Indian, adolescent, youth

## Abstract

**Introduction:**

Many causes of cancer related morbidity and mortality can be traced back to childhood behaviors. The culmination of cancer related risk and protective factors impacting the health and wellbeing of American Indian youth is unknown. The aim of this Mixed Studies Review was to identify cancer related risk and protective factors among American Indian youth. Results will be shared with Tribal communities to inform surveillance efforts.

**Methods:**

A Mixed Studies Review process was deemed most appropriate for the search process and data collection. Seven databases were included in the search along with 3 databases that were hand searched. Google Scholar and Google power searching were also conducted. Covidence was utilized for abstract and full-text review. Out of 1,512 articles, 75 articles were included for review and data from each article was sorted out into the levels of the social ecological model.

**Results:**

After extracting significant cancer related risk and protective factors from the 75 relevant articles, cancer related themes were identified at the individual, relationship (family and non-family), community, institutional, and cultural levels of the social ecological model. It was observed that the risk and protective factor profile for substance use spanned all levels of the social ecological model, whereas physical health-diet indicators and sexual health indicators did not. Most articles (*n* = 58, 77%) focused on substance use-related risk and protective factors.

**Discussion:**

The method that was used for this study can be utilized by other professionals researching risk and protective factors impacting the health and wellbeing of American Indian youth for a multitude of health outcomes. Tribal communities will be able to use the results from our literature review to inform the creation of a community specific data collection tool focused on cancer related risk and protective factors. Upon completion of the overarching research, results will be shared with the community and can be used to inform ongoing surveillance efforts, influence priorities for intervention and education work, and inform the management of resources. The continuation of community informed and driven research with Tribal communities is essential to the health and wellbeing of Tribal Nations as community grounded research is limited.

## Introduction

Many causes of cancer related morbidity and mortality can be traced back to childhood behaviors (1). The framework for the current study stems from the Youth Risk Behavior Surveillance System (YRBSS), which monitors health-related behaviors among youth and young adults. These health-related behaviors are categorized into six themes which include unintentional injuries and violence, sexual health behaviors, alcohol and other drug use, tobacco use, dietary behaviors, and physical activity behaviors (1). The categories that are cancer related are sexual health behaviors, substance use, and dietary and physical activity behaviors. The YRBSS is designed to determine the prevalence of health behaviors and assess changes over time. Although the YRBSS disseminates useful results for youth across the nation, it is not representative for Tribal nations. Currently there are only two representative Tribal government surveys included, Cherokee Nation and Winnebago Tribe (2). There are 574 federally recognized tribes in the United States (3), so surveys that are representative are needed for most of the Tribal Nations. As a result, the risk and protective profiles for these cancer related categories is unknown for American Indian youth. Therefore, a review of the literature was necessary to identify the risk and protective factors associated with these cancer related categories (substance use, diet and physical health, sexual health, etc.) in Tribal communities.

**Figure 1:**
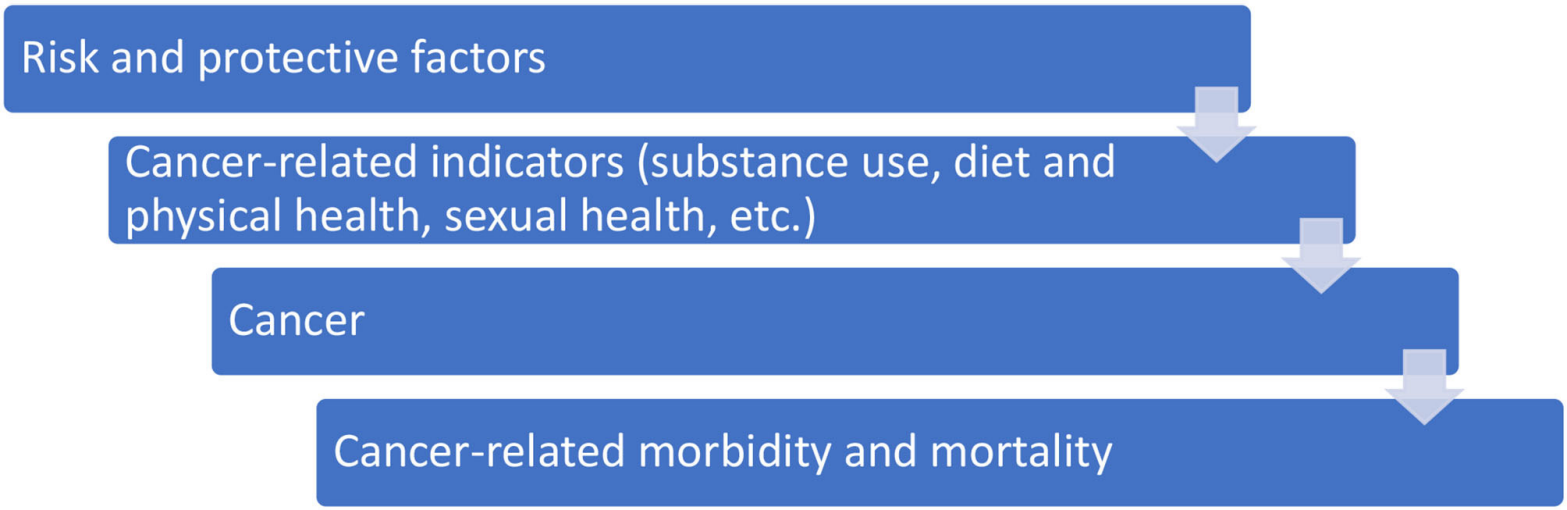
Concept model for cancer related risk and protective factors.

The purpose of this literature review was to identify common risk and protective factors for cancer related indicators (substance use, sexual health, diet, and physical health, etc.) that are likely to result in cancer related morbidity and mortality over the lifespan for American Indian youth (see Figure 1). This literature review is part of a larger research project that is focusing on developing and administering a community informed data collection tool with one Tribal community. The goal of the overarching research proposal is to determine the prevalence of community level cancer-related risk and protective factors among Tribal youth by creating and administering a community informed data collection tool.

American Indian populations suffer disproportionately from cancer compared to other races and ethnicities in the nation. According to the United States Cancer Statistics (USCS) in 2018, 10,019 new cases of cancer were reported for American Indian and Alaska Native people, and 3,502 American Indian and Alaska Native people died of cancer (4). In 2018, for every 100,000 American Indian and Alaska Native people, 259 new cancer cases were reported and 98 died of cancer (4). According to Espey et al., cancer was the leading cause of death for Native women and the second leading cause of death for Native men between the years of 1999–2009 (5). Cancer incidence rates vary by region for American Indians, whereas rates among non-hispanic Whites do not. Wiggens et al. explains that cancer rates for American Indians are the highest in Northern and Southern Plains in the United States (6). For all regions combined, the cancer related death rates for American Indians were nearly 50% greater than rates for Whites (6).

A multitude of factors impact the health and wellbeing of American Indian youth and many of these factors are known to lead to cancer over the lifespan. According to the Department of Health and Human Services, “Cigarette smoking increases the risk of... cancers of the lung, larynx, oral cavity, pharynx, pancreas, and cervix...” (7, 8). Smokeless tobacco increases the risk of developing cancer of the oral cavity and cigars increase the risk of developing lung, oral, and pharyngeal cancer (9–12). Lung cancer is one of the leading causes of cancer diagnosis and death for American Indians in the Northern Plains (13). American Indians in the Northern Plains also have a Larynx Cancer Death rate that is 2.5 times higher compared to Whites (13). Nutrition and physical activity-related health conditions specific to cancer include breast and colorectal cancer (14). Along with lung cancer, breast and colorectal cancer are also leading causes of diagnosis and death American Indians in the Northern Plains (13). “There is probable evidence to suggest that dietary patterns with higher intakes of fruits and vegetables are associated with a decreased risk for some types of cancer...” (15–17). Consistent regular physical activity decreases the risk of... some types of cancer (i.e., breast and colorectal), and premature death (18).

To our knowledge, a comprehensive community specific data collection tool that assesses the risk and protective factors for cancer related indicators (substance use, sexual health, diet, and physical health, etc.) that is informed by both the literature and the community has not been developed. The results of this review will be used to educate the community regarding the national presentation of previous research utilizing the social ecological model. The social ecological model conceptualizes health broadly, focusing on the impact of multiple factors, as well as the interaction between these factors at multiple levels (19). Identifying modifiable factors and understanding the social ecological presentation of these factors more broadly and at the Tribal level can inform community efforts aimed at improving the health and wellbeing of American Indian youth. These activities will inform the process of developing a Tribally informed data collection tool. Once the tool is developed and administered to Tribal youth, the results from the overarching study will be shared with Tribal communities to inform ongoing surveillance efforts, aid in prioritization and decision making, and guide the development of health-related interventions.

## Methods

Prior to creating a community informed survey, a review of existing literature was necessary. This literature review examined quantitative and qualitative peer reviewed studies to identify risk and protective factors for cancer related indicators (substance use, sexual health, diet, and physical health, etc.) among American Indian youth aged 10–21. Findings are organized by levels of the social ecological model and categorized into three themes, substance use, sexual health indicators, and diet-physical health indicators (see Table 1).

**Table 1:** Risk and protective factor themes.

**SEM level**	**Cancer themes**	**Risk/protective factors**
Individual	Substance use (45) • Alcohol = 8 • Tobacco = 8 • Hard drugs = 3 • All = 26	**Risk factors**: stressful life events, prior substance use/early substance initiation **Protective factors**: connected to school, participation in extracurriculars
	Physical health-diet indicators (12)	**Risk factors**: screen time/TV viewing, consumption of junk food **Protective factors**: participation in sports team, participation in physical activity
	Sexual health indicators (9)	**Risk factors**: substance use and having been sexually abused **Protective factors**: involvement in extracurriculars and self-efficacy
Relationship (Non-family)	Substance use (26) • Alcohol = 5 • Tobacco = 4 • Hard drugs = 3 • All = 14	**Risk factors**: friends' substance use and substance use offers from friends and family friends **Protective factors**: supportive/positive friendships and having a role model
	Physical health-diet indicators (0)	**Risk factors**: none identified **Protective factors**: none identified
	Sexual health indicators (3)	**Risk factors**: none identified **Protective factors**: support from friends and having a role model
Relationship (family)	Substance use (27) • Alcohol = 7 • Tobacco = 3 • Hard drugs = 2 • All = 15	**Risk factors**: family substance use and lower family SES **Protective factors**: family connectedness and family norms that discourage substance use
	Physical health-diet indicators (9)	**Risk factors**: parental weight-related behaviors (sedentary, TV viewing, diet) and lower family SES **Protective factors**: Having physically active parents
	Sexual health indicators (7)	**Risk factors**: none identified **Protective factors**: feeling connected to family and family communication
Community	Substance use (9) • Alcohol = 2 • Tobacco = 2 • Hard drugs = 0 • All = 5	**Risk factors**: lack of opportunities and access to substances **Protective factors**: opportunities for prosocial involvement and positive social norms
	Physical health-diet indicators (1)	**Risk factors**: environmental risks **Protective factors**: none identified
	Sexual health indicators (0)	**Risk factors**: none identified **Protective factors**: none identified
Institutional	Substance use (5) • Alcohol = 2 • Tobacco = 0 • Hard drugs = 1 • All = 2	**Risk factors**: none identified **Protective factors**: clear rules at school
	Physical health-diet indicators (0)	**Risk factors**: none identified **Protective factors**: none identified
	Sexual health indicators (3)	**Risk factors**: none identified **Protective factors**: opportunities for extracurriculars and relationships formed at school
Cultural	Substance use (7) • Alcohol = 1 • Tobacco = 2 • Hard drugs = 0 • All = 4	**Risk factors**: ethnic dislocation and historical trauma **Protective factors**: cultural connectedness having strong cultural/ religious values
	Physical health-diet indicators (0)	**Risk factors**: none identified **Protective factors**: none identified
	Sexual health indicators (2)	**Risk factors**: none identified **Protective factors**: engagement with cultural activities and spiritual traditions

### Mixed Studies Review Criteria

A Mixed Studies Review (MSR) search process is best for topics that have a body of literature that includes quantitative, qualitative, and mixed methods studies. The authors utilized the Toolkit for Mixed Studies Review (20). MSR is a literature review approach in which studies are systematically identified, selected, appraised, and synthesized. The first step in a MSR is to formulate a question, either qualitative, quantitative, or both (20). For this search, we wanted to understand what significant and salient risk and protective factors were impacting cancer related indicators present among American Indian youth. Quantitative and qualitative studies were screened for inclusion. The inclusion and exclusion criteria can be found below (see section Inclusion and Exclusion Criteria). Next, we identified the sources of information where data could be found. This included multiple databases, Google Scholar and Google power searches. With these different sources, an exhaustive search of relevant documents was undertaken. Relevant studies were pulled from the different sources by the University of North Dakota, School of Medicine and Health Science's Research and Education Librarian (author Olson), who was responsible for search strategies and formulating the search phrase. After completing the search, relevant studies were selected by two reviewers and the Principal Investigator (author Nadeau) to reduce bias. The Principal Investigator, a social/behavioral epidemiologist, assessed the quality of selected studies and extracted the data. Lastly, the results were combined and interpreted.

### Search Phrase

The research team wanted to ensure that all aspects of literature were included in this search. To do this, the following databases were included in the search: PubMed, CINAHL, PsychInfo, ERIC, University of New Mexico Native Health Database, Google Scholar, and iPortal. Journal hand searches have also been conducted for the Journal of Indigenous Research, the American Indian and Alaska Native Mental Health Research Journal, and Journal AlterNative. Google Scholar and Google power searching were also conducted. The search conducted for Google Scholar and Google power searches consisted of viewing articles until there were two consecutive Google pages with no relevant articles. The search phrase that was utilized for all databases was (“risk factor” OR “supportive factor” OR “supportive mechanism” OR “protective factor” OR “protective mechanism”) AND (“Native American” OR “American Indian” OR “Alaska Native”) AND (adolescent OR teen OR youth OR “young adult”). This phrase was inclusive of all relevant subjects that the team wanted to focus on. The focus of this review was on identifying cancer related risk and protective factors, so the word cancer was not included in the search phrases to avoid missing relevant articles. The Google Scholar and Google power search phrases were similar, but included “site:.gov”, “site:.edu”, and “site:.org” in the beginning of the phrase to only show these types of web pages.

### Inclusion and Exclusion Criteria

Inclusion and exclusion criteria were created. To be included for review, studies had to be published later than January 1st, 1970, and had to be conducted in the contiguous United States. In addition, studies needed to include significant and/or salient risk and/or protective factors for cancer related indicators “A protective factor can be defined as a characteristic at the biological, psychological, family, or community (including peers and culture) level that is associated with a lower likelihood of problem outcomes or that reduces the negative impact of a risk factor on problem outcomes” (21). Risk factors, are, “characteristics at the biological psychological, family, community, or cultural level that precedes and is associated with a higher likelihood of problem outcomes” (21). Lastly, most of the population included in each study had to be within the age range of 10–21 years. For example, if the age range was 16–25, and most of the study population fell within the age range of 10–21 years, the study would be included. Similar work being conducted in the field defined adolescence as 10–21 to be as inclusive as possible, as no standard age definition currently exists for the adolescent life stage (22–24).

### Covidence

**Figure 2:**
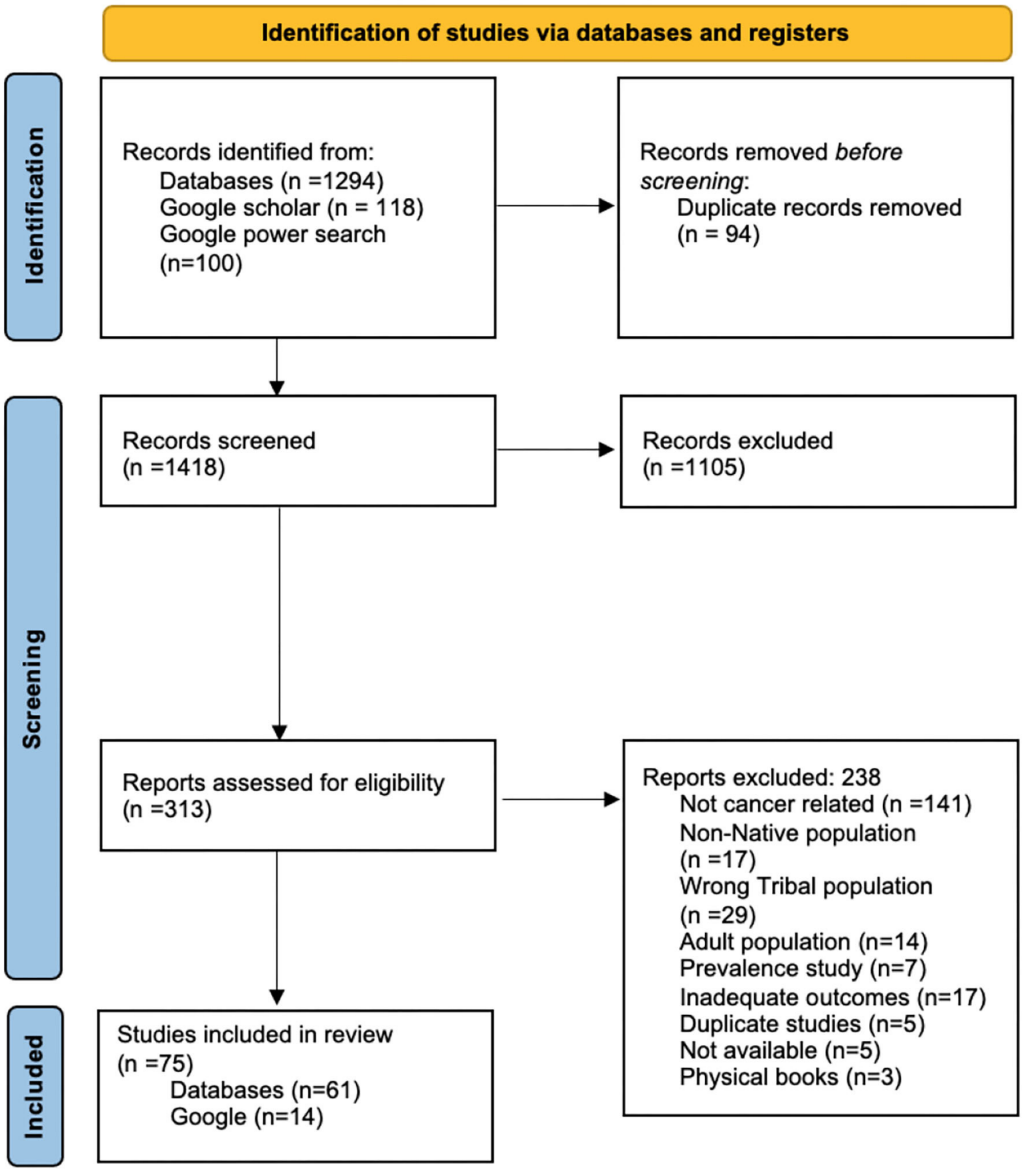
PRISMA model.

Covidence, a web-based software program, was utilized for abstract and full-text review. Articles that were found in the search were uploaded into the Covidence platform where researchers could read the abstracts and full texts on the web page. Both master level students read through abstracts and gave the studies a “yes”, “no”, or “maybe” vote. “Yes” and “maybe” votes were moved onto full text review. Conflicts were resolved by the Principal Investigator. The full text review differed slightly, as reviewers had to provide a reason for exclusion (see Figure 2). One thousand five hundred twelve articles were imported for screening and 94 duplicate articles were removed. One thousand four hundred eighteen studies were screened by title and abstract and 1,105 studies were deemed irrelevant. Three hundred thirteen full-text articles were assessed for eligibility, 238 studies were excluded. One hundred forty-one of these were not cancer related, 17 were not American Indian populations, 29 included the wrong Tribal population, 14 included adults, 7 were prevalence studies, 17 contained wrong outcomes (no salient outcomes), 5 were duplicate studies, 5 were not accessible, and 3 were physical books. With this, 75 studies were included for data extraction, 61 from databases and 14 from Google.

### Data Extraction Process

The data was pulled from the 75 relevant articles by the research team (see Appendix in Supplementary Material). Significant and salient results were gathered and put into a spreadsheet which was sorted out by levels of the social ecological model [individual, relationship (family and non-family), community, institutional, and cultural]. Then, results were thematically combined and tallied to find which risk and protective factors were most prevalent. These results are shown in Table 1 and described in the results section.

## Results

After extracting significant and salient cancer related risk and protective factors from relevant articles, cancer related themes were identified at the individual, relationship (family and non-family), community, institutional, and cultural levels of the social ecological model (see Table 1).

### Individual Level

At the individual level, the most prevalent risk factors for substance use include stressful life events and prior substance use or early substance initiation. The protective factor for substance use that was most prevalent was being connected to school and participation in extracurriculars. When looking at physical health-diet indicators at the individual level, the prominent risk factor was TV viewing/screen time and consumption of junk food. The significant protective factors were participation in a sports team or participating in physical activity in general. The risk and protective factors that were significant at the individual level for sexual health indicators were substance use and having been sexually abused, and the significant protective factors are involvement in extracurricular activities and self-efficacy.

### Relationship Level (Non-family)

The most prominent risk and protective factors found at the non-family relationship level for substance use are friends' substance use and receiving substance use offers from friends and family friends and the protective factors are supportive and positive friendships and having a role model. No risk or protective factors for physical health-diet indicators were found at this level. No risk factors were identified for sexual health indicators at the relationship level. The significant protective factors for sexual health indicators at the relationship level are support from friends and having a role model.

### Relationship Level (Family)

At the family relationship level, the most significant risk factors for substance use are family substance use and a lower household socioeconomic status (SES). The significant protective factors are family connectedness and family norms that discourage substance use. The most prominent risk factor found at the family relationship level for physical health-diet indicators were parental weight-related behaviors (such as TV viewing, sedentary behaviors, and diet), and lower family SES. The significant protective factor identified was having physically active parents. No risk factors were found for sexual health indicators at the family relationship level. The prominent protective factors found at the family relationship level for sexual health indicators were feeling connected to family and family communication.

### Community Level

The community level risk factors for substance use that were identified were lack of opportunities and access to alcohol and cigarettes. The significant protective factors for the community level were opportunities for prosocial involvement and positive social norms. Risk factors that were identified for physical health-diet indicators at the community level were environmental risks. No protective factors for physical health-diet indicators at the community level were found. No risk or protective factors were found at the community level for sexual health indicators.

### Institutional Level

No significant risk factors were found at the institutional level for substance use, physical health-diet indicators, or sexual health indicators. The significant protective factors identified for substance use were having clear rules at school. No protective factors were found for physical health-diet indicators at the institutional level. The protective factors for sexual health indicators at the institutional level were opportunities for extracurriculars and relationships formed at school.

### Cultural Level

Significant risk factors that were found at the cultural level for substance use were ethnic dislocation and historical trauma. The prominent protective factors for substance use at the cultural level were cultural connectedness having strong cultural and/or religious values. No risk or protective factors were identified at the cultural level for physical health-diet indicators. No risk factors were identified for sexual health indicators at the cultural level. The significant protective factors for sexual health indicators at the cultural level were engagement with cultural activities and spiritual traditions.

**Figure 3:**
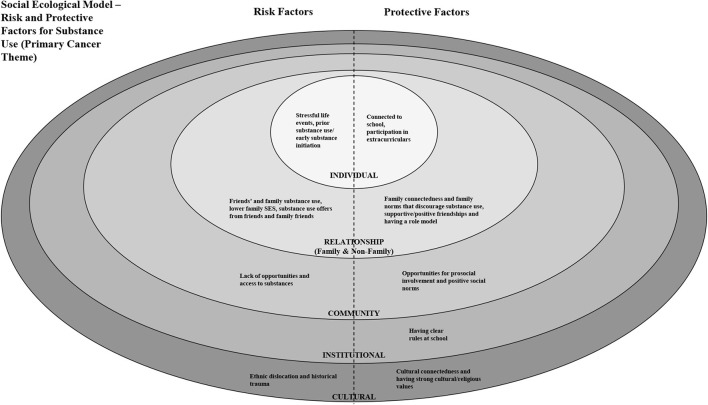
Social ecological model - risk and protective factors for substance use (primary cancer theme).

The research team explored the presentation of significant and salient findings at the individual, relationship (non-family), relationship (family), community, institutional, and cultural levels. No cancer related themes were identified at the policy level. Substance use presented as a key cancer related indicator, with the presence of associated risk and protective factors, at all levels of the social ecological model (see Figure 3). Substance use indicators were also identified as risk factors for substance use at the individual, relationship (non-family) and relationship (family) level. For example, at the individual level, risk factors for alcohol use include using tobacco and marijuana (25). At the relationship level, peer and family substance use are risk factors for substance use. Substance use was also a risk factor for sexual health indicators at the individual level and physical health-diet indicators at the relationship (non-family) level.

## Strengths and Limitations

This study had limitations which included not having full access to all available databases. Despite having a broad search phrase and the utilization of multiple databases and Google Scholar, there may have been relevant articles that were missed in our search. Of further consideration, the majority of articles (*n* = 58, 77%) focused on substance use-related risk and protective factors, indicating limited research focused on physical health-diet and sexual health indicators. Furthermore, as mentioned by Mackin et al. in 2012, additional risk and protective factors for cancer may exist for American Indian youth, that are not yet examined or published (26). And finally, there are 574 federally recognized tribes in the United States (3). Each tribe has its own culture, language, practices, and resources. Given these differences and the presentation of cancer varying from tribe to tribe, the risk and protective factors presented here may not be generalizable to all tribes and the translation of pertinent findings may vary at the community level.

The strengths of this study included having multiple reviewers, the use of Covidence, use of a multitude of databases and utilization of Google Scholar and Google power searches. The use of Google broadens the search so that the inclusion of marginalized voices, or perspectives not included in traditional academic searches is more likely. Without the utilization of Google power searches and Google Scholar, researchers are potentially missing out on salient articles that can be included to enhance research studies. Exploring multiple sources expanded the breadth of our search and diversified our search strategy. The use of multiple reviewers during our data extraction process and screening process is essential to minimizing biases and human error. Without the use of Google Scholar, 14 articles would not have been found even with the use of the broad search phrase that was applied in this study.

## Discussion

This is the first review of its kind. However, a similar review, conducted in 2018 by Hensen et al., explored protective factors across multiple health outcomes for AIAN adolescents. Henson et al. discusses the effects of protective factors on positive social and health outcomes among AI/AN youth and touches on topics including substance use, mental health, and delinquent behavior (27). The authors found that protective factors span multiple levels of the social ecological model and explain how findings could guide strength-based health promotion and prevention programming for AI/AN youths (27). Henson et al. found that protective impacts of culture also span all levels of the social ecological model and recommend the need for better data collection tools that measure cultural factors and incorporate the Tribal communities' views regarding research priorities as well as factors that impact the health and wellbeing of Tribal youth (27).

Although fewer (*n* = 17, 23%) articles focused on physical health-diet and sexual health indicators, meaningful findings arose from the search. Engagement with cultural activities and spiritual traditions presented as protective factors for sexual health indicators. Another protective factor for sexual health indicators is feeling connected to family and family communication. For physical health diet indicators, having physically active parents presented as a protective factor. Tribal people are very family oriented and value family connectedness as part of their culture. As previously mentioned, cultural indicators span the social ecological model. Promoting culture and initiatives grounded in cultural values would be a meaningful way for Tribal communities to advocate, support and engage in protective factor rich environments and positively impact the health of youth at multiple levels of community.

A summary table containing all substance use related articles (n = 22) and related protective factors is shown in Supplementary Table 1 and a social ecological model illustrating significant risk and protective factors for substance use is shown in Figure 3. This information can be used to inform Tribally driven/informed data collection efforts. Initiatives focused on reducing substance use/abuse would benefit from focusing on risk and protective factors that span the social ecological model.

### Future Directions

For this research, a Mixed Studies Review was deemed most adequate. This process can be utilized by other professionals researching various study types (i.e., quantitative, qualitive, and mixed) for risk and protective factors impacting the health and wellbeing of American Indian youth for a multitude of health outcomes. A multitude of factors contribute to the high rates of cancer in Indian Country in addition to these well-known cancer indicators including the impacts resulting from intergenerational trauma, barriers to prevention and care due to high rates of poverty, lack of access to healthy foods and underfunding (28). Our review identified a multitude of cancer related risk and protective factors, with the majority being identified at the individual, family relationship level and non-family relationship level.

The results from this study outline the risk and protective factors that can be found in American Indian communities in the contiguous United States. Tribal communities will be able to use the results from our literature review to inform the creation of a community specific data collection tool focused on cancer related risk and protective factors. Upon completion of the overarching research, results will be shared with the community which will inform ongoing surveillance efforts, influence priorities for intervention and education work, and inform the management of resources. The continuation of community informed and driven research with Tribal communities is essential to the health and wellbeing of Tribal Nations as community grounded research is limited.

Approximately 30% of what impacts our health can be attributed to health behavior. However, the physical environment along with social and economic factors impacts ~50% of our health. More research is needed to assess community, policy and cultural impacts on youth health and wellness. To properly assess AI youth health and wellbeing, we must look at conditions that create or limit opportunity. Doing so will provide us with the important perspective for understanding both the nature and the sources of disparate health outcomes and will guide us to viable and effective solutions (28).

## Data Availability Statement

The original contributions presented in the study are included in the article/Supplementary Material, further inquiries can be directed to the corresponding author/s.

## Author Contributions

MN, DO, and KS: study conception and design of the work. MN, DO, KW, and VF: data collection. MN, KW, and VF: data analysis and interpretation of results. MN and KW: draft manuscript preparation. All authors reviewed the results and approved the final version of the manuscript to be published.

## Funding

Research reported in this publication was supported by the National Institute of General Medical Sciences of the National Institutes of Health under Award Number U54GM128729.

## Author Disclaimer

The content is solely the responsibility of the authors and does not necessarily represent the official views of the National Institutes of Health.

## Conflict of Interest

The authors declare that the research was conducted in the absence of any commercial or financial relationships that could be construed as a potential conflict of interest.

## Publisher's Note

All claims expressed in this article are solely those of the authors and do not necessarily represent those of their affiliated organizations, or those of the publisher, the editors and the reviewers. Any product that may be evaluated in this article, or claim that may be made by its manufacturer, is not guaranteed or endorsed by the publisher.
