# Supplementary material for: Identification of Cancer Related Risk and Protective Factors for American Indian Youth: A Mixed Studies Review
Source: Front Public Health. 2022 Apr 25;10:828776. doi: 10.3389/fpubh.2022.828776 (PMC9081341; doi:10.3389/fpubh.2022.828776)
Supplement: Supplementary file 1 [file Data_Sheet_1.pdf]

Appendix
Articles (n=75) Included in Extraction

1. Baldwin, J. A., Brown, B. G., Wayment, H. A., Nez, R. A., & Brelsford, K. M. (2011). Culture and context: Buffering the relationship between stressful life events and risky behaviors in American Indian youth. *Substance Use and Misuse*, *46*(11), 1380–1394. https://doi.org/10.3109/10826084.2011.592432

2. Beebe, L. A., Vesely, S. K., Oman, R. F., Tolma, E., Aspy, C. B., & Rodine, S. (2008). Protective assets for non-use of alcohol, tobacco and other drugs among urban American Indian youth in Oklahoma. *Maternal and Child Health Journal*, *12*(1), 82–90. https://doi.org/10.1007/s10995-008-0325-5

3. Binion, A., Miller, C. D., Beauvais, F., & Oetting, E. R. (1988). Rationales for the use of alcohol, marijuana, and other drugs by eighth-grade native american and anglo youth. *Substance Use and Misuse*, *23*(1), 47–64. https://doi.org/10.3109/10826088809027490

4. Cameron, LA. (1999). Understanding alcohol abuse in American Indian/Alaskan Native youth. *Pediatric Nursing*, *25*(3), 297–300.

5. Cary, M. (2005). *Cultural Connectedness and Regular Smoking Among a Cohort of Urban American Indian Youth*.

6. DeLong, A., Larson, N., Story, M., Neumark-Sztainer, D., Weber-Main, A., & Ireland, M. (2008). Factors associated with overweight among urban American Indian adolescents: findings from Project EAT. *Ethn Dis.*, *18*(3), 317–323. https://web-a-ebscohost-com.ezproxylr.med.und.edu/ehost/detail/detail?vid=0&sid=9c8085ff-481f-4044-999d-34960da2ba55%40sdc-v-sessmgr01&bdata=JnNpdGU9ZWhvc3QtbGl2ZQ%3d%3d#AN=105556042&db=ccm

7. Devries, K. M., Free, C. J., Morison, L., & Saewyc, E. (2009). Factors associated with the sexual behavior of Canadian aboriginal young people and their implications for health promotion. *American Journal of Public Health*, *99*(5), 855–862. https://doi.org/10.2105/AJPH.2007.132597

8. Devries, K. M., Free, C. J., Morison, L., & Saewyc, E. (2009). Factors Associated with Pregnancy and STI among Aboriginal students in British Columbia. *Canadian Journal of Public Health*, *100*(3), 226–230. https://doi.org/10.1007/bf03405546

9. Dickens, D. D., Dieterich, S. E., Henry, K. L., & Beauvais, F. (2012). School bonding as a moderator of the effect of peer influences on alcohol use among American Indian adolescents. *Journal of Studies on Alcohol and Drugs*, *73*(4), 597–603. https://doi.org/10.15288/jsad.2012.73.597

10. Eitle, D., & Eitle, T. (2013). Methamphetamine use among rural white and native American adolescents: An application of the stress process model. *Journal of Drug Education*, *43*(3), 203–221. https://doi.org/10.2190/DE.43.3.a

11. Eitle, T. M. N., Johnson-Jennings, M., & Eitle, D. J. (2013). Family structure and adolescent alcohol use problems: Extending popular explanations to American Indians. *Social Science Research*, *42*(6), 1467–1479. https://doi.org/10.1016/J.SSRESEARCH.2013.06.007

12. Fairman, B. J., Furr-Holden, C. D., & Johnson, R. M. (2019). When Marijuana Is Used before Cigarettes or Alcohol: Demographic Predictors and Associations with Heavy Use, Cannabis Use Disorder, and Other Drug-related Outcomes. *Prevention Science*, *20*(2), 225–233. https://doi.org/10.1007/s11121-018-0908-3

13. Federman, E. B., Costello, E. J., Angold, A., Farmer, E. M. Z., & Erkanli, A. (1997). Development of substance use and psychiatric comorbidity in an epidemiologic study of white and American Indian young adolescents: The Great Smoky Mountains Study. *Drug and Alcohol Dependence*, *44*(2–3), 69–78. https://doi.org/10.1016/S0376-8716(96)01317-8

14. Freedman, D. S., Serdula, M. K., Percy, C. A., Ballew, C., & White, L. (1997). Obesity, levels of lipids and glucose, and smoking among Navajo adolescents. *Journal of Nutrition*, *127*(10), 2120–2127. https://doi.org/10.1093/jn/127.10.2120s

15. Friese, B., Grube, J. W., Seninger, S., Paschall, M. J., & Moore, R. S. (2011). Drinking behavior and sources of alcohol: Differences between Native American and White youths. *Journal of Studies on Alcohol and Drugs*, *72*(1), 53–60. https://doi.org/10.15288/jsad.2011.72.53

16. Garrett, B. A., Livingston, B. J., Livingston, M. D., & Komro, K. A. (2017). The Effects of Perceived Racial/Ethnic Discrimination on Substance Use Among Youths Living in the Cherokee Nation. *Journal of Child and Adolescent Substance Abuse*, *26*(3), 242–249. https://doi.org/10.1080/1067828X.2017.1299656

17. Gilchrist, L. D., Schinke, S. P., Trimble, J. E., & Cvetkovich, G. T. (1987). Skills enhancement to prevent substance abuse among american indian adolescents. *Substance Use and Misuse*, *22*(9), 869–879. https://doi.org/10.3109/10826088709027465

18. Greene, K. M., Eitle, D., & Eitle, T. M. N. (2018). Developmental Assets and Risky Sexual Behaviors Among American Indian Youth. *Journal of Early Adolescence*, *38*(1), 50–73. https://doi.org/10.1177/0272431615596427

19. Greene, K. M., Eitle, T. M. N., & Eitle, D. (2014). Adult social roles and alcohol use among American Indians. *Addictive Behaviors*, *39*(9), 1357–1360. https://doi.org/10.1016/j.addbeh.2014.04.024

20. Griese, E. R., Kenyon, D. Y. B., & McMahon, T. R. (2016). Identifying sexual health protective factors among northern plains American Indian youth: An ecological approach utilizing multiple perspectives. *American Indian and Alaska Native Mental Health Research*, *23*(4), 16–43. https://doi.org/10.5820/aian.2304.2016.16

21. Guttmannova, K., Wheeler, M. J., Hill, K. G., Evans-Campbell, T. A., Hartigan, L. A., Jones, T. M., Hawkins, J. D., & Catalano, R. F. (2017). ASSESSMENT OF RISK AND PROTECTION IN NATIVE AMERICAN YOUTH: STEPS TOWARD CONDUCTING CULTURALLY RELEVANT, SUSTAINABLE PREVENTION IN INDIAN COUNTRY. *Journal of Community Psychology*, *45*(3), 346–362. https://doi.org/10.1002/jcop.21852

22. Hawkins, E. H., Marlatt, G. A., & Cummins, L. H. (2004). Preventing Substance Abuse in American Indian and Alaska Native Youth: Promising Strategies for Healthier Communities. In *Psychological Bulletin* (Vol. 130, Issue 2, pp. 304–323). https://doi.org/10.1037/0033-2909.130.2.304

23. Henson, M., Sabo, S., Trujillo, A., & Teufel-Shone, N. (2017). Identifying Protective Factors to Promote Health in American Indian and Alaska Native Adolescents: A Literature Review. *Journal of Primary Prevention*, *38*(1–2), 5–26. https://doi.org/10.1007/s10935-016-0455-2

24. Hirchak, K., Amiri, S., Espinoza, J., Herron, J., Hernandez-Vallant, A., Cloud, V., & Venner, K. (2021). TRENDS IN NON-MEDICAL PRESCRIPTION OPIOID USE AMONG URBAN AND RURAL AMERICAN INDIAN AND ALASKA NATIVE YOUTH RESIDING IN NEW MEXICO: 2013-2017. *Am Indian Alsk Native Ment Health Res.*, *28*(1), 1–16. www.coloradosph.cuanschutz.edu/caianh

25. Jollie-Trottier, T., Holm, J. E., & McDonald, J. D. (2009). Correlates of overweight and obesity in American Indian children. *Journal of Pediatric Psychology*, *34*(3), 245–253. https://doi.org/10.1093/jpepsy/jsn047

26. Karen Chan Osilla, Heather S. Lonczak, Patricia D. Mail, Mary E. Larimer, & G. Alan Marlatt. (2008). Regular Tobacco Use Among American Indian and Alaska Native Adolescents. *Journal of Ethnicity in Substance Abuse*, *6*(3–4), 143–153. https://doi.org/10.1300/J233V06N03_06

27. Kaufman, C. E., Desserich, J., Big Crow, C. K., Holy Rock, B., Keane, E., & Mitchell, C. M. (2007). Culture, context, and sexual risk among Northern Plains American Indian Youth. *Social Science & Medicine*, *64*(10), 2152–2164. https://doi.org/10.1016/J.SOCSCIMED.2007.02.003

28. Komro, K. A., Livingston, M. D., Garrett, B. A., & Boyd, M. L. (2016). Similarities in the etiology of alcohol use among native american and non-native young women. *Journal of Studies on Alcohol and Drugs*, *77*(5), 782–791. https://doi.org/10.15288/jsad.2016.77.782

29. Komro, K. A., Livingston, M. D., Wagenaar, A. C., Kominsky, T. K., Pettigrew, D. W., Garrett, B. A., Boyd, B. J., Boyd, M. L., Livingston, B. J., Lynne, S. D., Molina, M. M., Merlo, L. J., & Tobler, A. L. (2017). Multilevel prevention trial of alcohol use among American Indian and white high school students in the Cherokee nation. *American Journal of Public Health*, *107*(3), 453–459. https://doi.org/10.2105/AJPH.2016.303603

30. Kulis, S., Hodge, D. R., Ayers, S. L., Brown, E. F., & Marsiglia, F. F. (2012). Spirituality and religion: Intertwined protective factors for substance use among urban American Indian youth. *American Journal of Drug and Alcohol Abuse*, *38*(5), 444–449. https://doi.org/10.3109/00952990.2012.670338

31. Kulis, S. S., Jager, J., Ayers, S. L., Lateef, H., & Kiehne, E. (2016). Substance Use Profiles of Urban American Indian Adolescents: A Latent Class Analysis. *Substance Use and Misuse*, *51*(9), 1159–1173. https://doi.org/10.3109/10826084.2016.1160125

32. Kulis, S., Okamoto, S. K., Rayle, A. D., & Sen, S. (2006). Social contexts of drug offers among American Indian youth and their relationship to substance use: An exploratory study. *Cultural Diversity and Ethnic Minority Psychology*, *12*(1), 30–44. https://doi.org/10.1037/1099-9809.12.1.30

33. Lee, C. T., Rose, J. S., Engel-Rebitzer, E., Selya, A., & Dierker, L. (2011). Alcohol dependence symptoms among recent onset adolescent drinkers. *Addictive Behaviors*, *36*(12), 1160–1167. https://doi.org/10.1016/j.addbeh.2011.07.014

34. LeMaster, P. L., Connell, C. M., Mitchell, C. M., & Manson, S. M. (2002). Tobacco use among American Indian adolescents: Protective and risk factors. *Journal of Adolescent Health*, *30*(6), 426–432. https://doi.org/10.1016/S1054-139X(01)00411-6

35. Lonczak, H. S., Fernandez, A., Austin, L., Marlatt, G. A., & Donovan, D. M. (2007). Family structure and substance use among American Indian youth: A preliminary study. *Families, Systems and Health*, *25*(1), 10–22. https://doi.org/10.1037/1091-7527.25.1.10

36. Lowe, J., Liang, H., Riggs, C., Henson, J., & Elder, T. (2012). Community Partnership to Affect Substance Abuse among Native American Adolescents. *Am J Drug Alcohol Abuse*, *38*(5), 450–455. https://doi.org/10.3109/00952990.2012.694534

37. Lynch, W. C., Heil, D. P., Wagner, E., & Havens, M. D. (2007). Ethnic differences in BMI, weight concerns, and eating behaviors: Comparison of Native American, White, and Hispanic adolescents. *Body Image*, *4*(2), 179–190. https://doi.org/10.1016/j.bodyim.2007.01.001

38. Mail, P. D. (1996). Cultural Orientation and Positive Psychological Status as Protective Factors Against Problem Behaviors in Southwestern American Indian Adolescents. *Dissertation Abstracts International Section A: Humanities and Social Sciences*, *58*(1A). https://web-b-ebscohost-

com.ezproxylr.med.und.edu/ehost/detail/detail?vid=0&sid=2031bc85-7a0d-42b0-b82c-eb12364fffd4%40pdc-v-sessmgr02&bdata=JnNpdGU9ZWhvc3QtbGl2ZQ%3d%3d#AN=1997-95013-114&db=psyh

39. Marsiglia, F. F., Nieri, T., & Stiffman, A. R. (2006). HIV/AIDS Protective Factors among Urban American Indian Youths. *Journal of Health Care for the Poor and Underserved*, *17*(4), 745. https://doi.org/10.1353/HPU.2006.0128

40. Martinez, M. J., Ayers, S. L., Kulis, S., & Brown, E. (2015). The Relationship Between Peer, Parent, and Grandparent Norms and Intentions to Use Substances for Urban American Indian Youths. *Journal of Child and Adolescent Substance Abuse*, *24*(4), 220–227. https://doi.org/10.1080/1067828X.2013.812529

41. Merritt, L. (2018). Exploring the Association of Victimization and Alcohol and Marijuana Use among American Indian Youth Living on or Near Reservations: A Mixed Methods Study. *Dissertations and Theses*. https://doi.org/10.15760/etd.6280

42. Miller, C. L., Pearce, M. E., Moniruzzaman, A., Thomas, V., Christian, W., Schechter, M. T., & Spittal, P. M. (2011). The Cedar Project: Risk factors for transition to injection drug use among young, urban Aboriginal people. *CMAJ*, *183*(10), 1147–1154. https://doi.org/10.1503/cmaj.101257

43. Mitchell, C. M., Kaufman, C. E., Beals, J., Bauduy, S., Bell, C. A. E., Crow, C. B., Buchwald, D., Cottier, N., Dethlefsen, A. D., Frederick, A. W., Keane, E., Hubing, S., Murphy, N., Sam, A., Settlemire, J., Truel, J., & Dress, F. W. (2004). Equifinality and multifinality as guides for preventive interventions: HIV risk/protection among American Indian young adults. *Journal of Primary Prevention*, *25*(4), 491–510. https://doi.org/10.1023/B:JOPP.0000048114.49642.b2

44. Mitchell, C. M., Beals, J., Kaufman, C. E., Bauduy, S., Bell, C. A. E., Big Crow, C. K., Buchwald, D., Cottier, N., Dethlefsen, A. D., Frederick, A. W., Keane, E. M., Hubing, S., Murphy, N., Sam, A., Settlemire, J., Truel, J., & Dress, F. W. (2006). Alcohol use, outcome expectancies, and HIV risk status among American Indian youth: A latent growth curve model with parallel processes. *Journal of Youth and Adolescence*, *35*(5), 729–740. https://doi.org/10.1007/s10964-006-9103-0

45. Moilanen, K. L., Markstrom, C. A., & Jones, E. (2014). Extracurricular Activity Availability and Participation and Substance Use Among American Indian Adolescents. *J Youth Adolesc*, *43*(3), 454–469. https://doi.org/10.1007/s10964-013-0088-1

46. Moncher, M. S., Holden, G. W., & Trimble, J. E. (1990). Substance Abuse Among Native-American Youth. *Journal of Consulting and Clinical Psychology*, *58*(4), 408–415. https://doi.org/10.1037/0022-006x.58.4.408

47. Nalven, T., Spillane, N. S., & Schick, M. R. (2020). Risk and protective factors for opioid misuse in American Indian adolescents. *Drug and Alcohol Dependence*, *206*. https://doi.org/10.1016/j.drugalcdep.2019.107736

48. Napoli, M., Marsiglia, F. F., & Kulis, S. (2003). Sense of belonging in school as a protective factor against drug abuse among native american urban adolescents. *Journal of Social Work Practice in the Addictions*, *3*(2), 25–41. https://doi.org/10.1300/J160v03n02_03

49. Ness, M., Barradas, D. T., Irving, J., & Manning, S. E. (2012). Correlates of overweight and obesity among American Indian/Alaska Native and Non-Hispanic White children and adolescents: National Survey of Children's Health, 2007. *Maternal and Child Health Journal*, *16*(2), 268–277. https://doi.org/10.1007/s10995-012-1191-8

50. Novins, D. K., & Barón, A. E. (2004). American Indian substance use: The hazards for substance use initiation and progression for adolescents aged 14 to 20 years. *Journal of the American Academy of Child and Adolescent Psychiatry*, *43*(3), 316–324. https://doi.org/10.1097/00004583-200403000-00013

51. Okwumabua, J. O., & Duryea, E. J. (1987). Age of onset, periods of risk, and patterns of progression in drug use among american indian high school students. *Substance Use and Misuse*, *22*(12), 1269–1276. https://doi.org/10.3109/10826088709027486

52. Patel, H., Chambers, R., Littlepage, S., Rosenstock, S., Richards, J., Lee, A., Slimp, A., Melgar, L., Lee, S., Susan, D., & Tingey, L. (2021). The association of parental monitoring and parental communication with sexual and substance use risk behaviors among Native American Youth. *Children and Youth Services Review*, *129*, 106171. https://doi.org/10.1016/J.CHILDYOUTH.2021.106171

53. Polley, D. C., Spicer, M. T., Knight, A. P., & Hartley, B. L. (2005). Intrafamilial correlates of overweight and obesity in African American and Native-American grandparents, parents, and children in rural Oklahoma. *Journal of the American Dietetic Association*, *105*(2), 262–265. https://doi.org/10.1016/j.jada.2004.11.004

54. Roski J, Perry CL, & McGovern PG. (2008). Psychosocial factors associated with alcohol use among young adolescent American Indians and Whites. *Journal of Child & Adolescent Substance Abuse*, *7*(2), 1–18. https://web-a-ebscohost-com.ezproxylr.med.und.edu/ehost/detail/detail?vid=0&sid=8ac7cb20-596f-4c70-b15a-057f3f47f1b1%40sessionmgr4008&bdata=JnNpdGU9ZWhvc3QtbGl2ZQ%3d%3d#AN=107287588&db=ccm

55. Sanchez-Way, R., & Johnson, S. (2000). Cultural Practices in American Indian Prevention Programs. *Juvenile Justice*, *7*. https://heinonline.org/HOL/Page?handle=hein.journals/juvejstc7&id=60&div=12&collection=journals

56. Schell, L. M., & Gallo, M. v. (2012). Overweight and obesity among North American Indian infants, children, and youth. *American Journal of Human Biology*, *24*(3), 302–313. https://doi.org/10.1002/ajhb.22257

57. Schinke, S. P., Singer, B., Cole, K., & Contento, I. R. (1996). Reducing cancer risk among Native American adolescents. *Preventive Medicine*, *25*(2), 146–155. https://doi.org/10.1006/pmed.1996.0040

58. Sittner, K. J. (2016). Trajectories of Substance Use: Onset and Adverse Outcomes Among North American Indigenous Adolescents. *Journal of Research on Adolescence*, *26*(4), 830–844. https://doi.org/10.1111/jora.12233

59. Soto, C., Baezconde-Garbanati, L., Schwartz, S. J., & Unger, J. B. (2015). Stressful life events, ethnic identity, historical trauma, and participation in cultural activities: Associations with smoking behaviors among American Indian adolescents in California. *Addictive Behaviors*, *50*, 64–69. https://doi.org/10.1016/j.addbeh.2015.06.005

60. Soto Navajo, C., Pueblo Jennifer Unger Kimberly Miller Ingrid Zeledon Victoria Telles Bryce Henderson VyVy Nguyen Cynthia Begay Hopi, J. M., Desirae Martinez Ramata Franklin, N., Johnson, C., Dickerson, D., Antony, V., Domaguin, D., Schweigman Oglala, K., Lakota, S., & Moerner, L. (2019). *ADDRESSING THE OPIOID CRISIS IN AMERICAN INDIAN & ALASKA NATIVE COMMUNITIES IN CALIFORNIA: A STATEWIDE NEEDS ASSESSMENT*.

61. Spillane, N. S., & Smith, G. T. (2007). A Theory of Reservation-Dwelling American Indian Alcohol Use Risk. *Psychological Bulletin*, *133*(3), 395–418. https://doi.org/10.1037/0033-2909.133.3.395

62. Spillane, N. S., Muller, C. J., Noonan, C., Goins, R. T., Mitchell, C. M., & Manson, S. (2012). Sensation-seeking predicts initiation of daily smoking behavior among American Indian high school students. *Addictive Behaviors*, *37*(12), 1303–1306. https://doi.org/10.1016/J.ADDBEH.2012.06.021

63. Spillane, N. S., Weyandt, L., Oster, D., & Treloar, H. (2017). Social contextual risk factors for stimulant use among adolescent American Indians. *Drug and Alcohol Dependence*, *179*, 167–173. https://doi.org/10.1016/j.drugalcdep.2017.06.032

64. Swaim, R. C., & Stanley, L. R. (2020). Predictors of Substance Use Latent Classes Among American Indian Youth Attending Schools on or Near Reservations. *American Journal on Addictions*, *29*(1), 27–34. https://doi.org/10.1111/ajad.12894

65. Thurman, P., & Green, V. A. (1997). American Indian Adolescent Inhalant Use. *Am Indian Alsk Native Ment Health Res.*, *8*(1), 24–40. https://web-b-ebscohost-com.ezproxylr.med.und.edu/ehost/detail/detail?vid=0&sid=601ffbd0-435d-422c-93b9-21532896c2fe%40pdc-v sessmgr03&bdata=JnNpdGU9ZWhvc3QtbGl2ZQ%3d%3d#AN=EJ615519&db=eric

66. Tingey, L., Cwik, M. F., Rosenstock, S., Goklish, N., Larzelere-Hinton, F., Lee, A., Suttle, R., Alchesay, M., Massey, K., & Barlow, A. (2016). Risk and protective factors for heavy binge alcohol use among American Indian adolescents utilizing emergency health services. *American Journal of Drug and Alcohol Abuse*, *42*(6), 715–725. https://doi.org/10.1080/00952990.2016.1181762

67. Tingey, L., Chambers, R., Rosenstock, S., Larzelere, F., Goklish, N., Lee, A., & Rompalo, A. (2018). Risk and Protective Factors Associated with Lifetime Sexual Experience Among Rural, Reservation-Based American Indian Youth. *Journal of Primary Prevention*, *39*(4), 401–420. https://doi.org/10.1007/s10935-018-0517-8

68. Tingey, L., Cwik, M., Goklish, N., Alchesay, M., Lee, A., Strom, R., Suttle, R., Walkup, J., & Barlow, A. (2012). Exploring binge drinking and drug use among American Indians: Data from adolescent focus groups. *American Journal of Drug and Alcohol Abuse*, *38*(5), 409–415. https://doi.org/10.3109/00952990.2012.705204

69. Unger, J. B., Shakib, S., Cruz, T. B., Hoffman, B. R., Pitney, B. H., & Rohrbach, L. A. (2003). Smoking behavior among urban and rural native American adolescents in California. *American Journal of Preventive Medicine*, *25*(3), 251–254. https://doi.org/10.1016/S0749-3797(03)00193-4

70. Unger, J. (2005). Family- and Peer-Related Risk and Protective Factors for Tobacco Use Among American Indian Adolescents in California. *Journal of Ethnicity in Substance Abuse*, *3*(4), 1–15. https://web-b-ebscohost-com.ezproxylr.med.und.edu/ehost/detail/detail?vid=0&sid=fc4e3e14-459f-44ed-bd3a-5ac6ef53d7e4%40sessionmgr101&bdata=JnNpdGU9ZWhvc3QtbGl2ZQ%3d%3d#AN=2005-09203-001&db=psyh

71. Weaver, H., & Jackson, K. (2012). Cancer risks and Native Americans: The “Healthy Living in Two Worlds” study. *Health Education Journal*, *71*(6), 688–698. https://doi.org/10.1177/0017896911422773

72. Whitesell, N. R., Asdigian, N. L., Kaufman, C. E., Big Crow, C., Shangreau, C., Keane, E. M., Mousseau, A. C., & Mitchell, C. M. (2014). Trajectories of Substance Use Among Young American Indian Adolescents: Patterns and Predictors. *Journal of Youth and Adolescence*, *43*(3), 437–453. https://doi.org/10.1007/s10964-013-0026-2

73. Whitesell, N. R., Kaufman, C. E., Keane, E. M., Crow, C. B., Shangreau, C., & Mitchell, C. M. (2012). Patterns of substance use initiation among young adolescents in a Northern Plains American Indian tribe. *American Journal of Drug and Alcohol Abuse*, *38*(5), 383–388. https://doi.org/10.3109/00952990.2012.694525

74. Whitesell, N. R., Beals, J., Mitchell, C. M., Manson, S. M., & Turner, R. J. (2009). Childhood exposure to adversity and risk of substance-use disorder in two American

Indian populations: The meditational role of early substance-use initiation. *Journal of Studies on Alcohol and Drugs*, *70*(6), 971–981. https://doi.org/10.15288/jsad.2009.70.971

75. Yu, M. S., & Stiffman, A. R. (2010). Positive family relationships and religious affiliation as mediators between negative environment and illicit drug symptoms in American Indian adolescents. *Addictive Behaviors*, *35*(7), 694–699. https://doi.org/10.1016/J.ADDBEH.2010.03.005
