# Supplementary material for: Identification of Cancer Related Risk and Protective Factors for American Indian Youth: A Mixed Studies Review
Source: Front Public Health. 2022 Apr 25;10:828776. doi: 10.3389/fpubh.2022.828776 (PMC9081341; doi:10.3389/fpubh.2022.828776)
Supplement: Supplementary file 2 [file Table_1.pdf]

Supplementary Table 1 - Summary table for all substance use related articles (n = 22) and related protective factors

Publication	Year	Population	Method	Instrument	Substance Type	Protective Factors for Substance Use
Beebe, Laura A.; Vesely, Sara K.; Oman, Roy F.; Tolma, Eleni; Aspy, Cheryl B.; Rodine, Sharon	2008	134 American Indian Adolescents Location: Oklahoma	Logistic regression analyses and interviews	Multi-racial/ethnicity study of youth assets and risk behaviors survey	Alcohol, tobacco, and other drug use	-Good health practices (exercise/nutrition) and use of time (religion)<br>- Having non-parental adult role models<br>- Family structure and two parent households
Dickens, Danielle D.; Dieterich, Sara E.; Henry, Kimberly L.; Beauvais, Fred	2012	2,582 Native students Location: United States (WA, OR, MT, AZ, ND, SD, MN, WI, NV, AL)	Survey	American Drug and Alcohol Survey	Alcohol use	-School bonding
Friese, Bettina; Grube, Joel W.; Seninger, Steve; Paschall, Mallie J.; Moore, Roland S.	2011	Total: 18,916 (1,416 Native youth) Location: Montana	Survey	2008 Prevention Needs Assessment Community Student Survey	Alcohol use	-Living in a county with a higher proportion of Native Americans
Gilchrist, Lewayne D.; Schinke, Steven Paul; Trimble, Joseph E.; Cvetkovich, George T.	1987	Total: 102 Native youth Location: Pacific Northwest	School-based survey	YRBSS	Alcohol, marijuana, and inhalant use	-Those who received skills enhancement intervention (knowledge) had lower rates of substance use
Greene, Kaylin M.; Eitle, Tamela Mc Nulty; Eitle, David	2014	927 Native youth Location: United States	Self-administered questionnaire	National Longitudinal Study of Adolescent Health	Alcohol use	-Becoming a parent and attending college
Guttmannova, Katarina; Wheeler, Melissa J.; Hill, Karl G.; Evans-Campbell, Teresa A.; Hartigan, Lacey A.; Jones, Tiffany M.; Hawkins, J. David; Catalano, Richard F.	2017	Total: 284,268 (5,095 Native youth) Location: United States	Survey	Communities That Care Youth Survey (CTC-YS)	Alcohol, marijuana, and cigarette use	-Social skills and belief in moral order<br>- Family attachment<br>- Opportunities for prosocial involvement and rewards for prosocial involvement
Hawkins, Elizabeth H.; Marlatt, G. Alan; Cummins, Lillian H.	2004	Literature review Location: United States			Substance use	-Self-efficacy in social relationships<br>- Participation in positive peer clusters

							- Stable and supportive relationships with pro-social adults<br>- Bonding to conventional society<br>- Community resources<br>- Participation in organized group activities<br>- Strong school bond<br>- Cultural involvement<br>- Involvement in religious activities
Henson, Michele; Sabo, Samantha; Trujillo, Aurora; Teufel-Shone, Nicolette	2017	Literature review Location: United States and Canada				Alcohol use, substance use, and tobacco use	-Wanting to be a role model<br>-Giving to others by contributing to the community<br>-Believing in one's value and potential<br>-Awareness of consequences<br>-Interconnection<br>-Having awareness of life goals<br>-Self-efficacy<br>-Involvement in sports team<br>-Participation in music<br>-Possessing college aspirations<br>-Positive adult role models<br>-Prosocial peer influence<br>-Close relationships with parents<br>-Parents acting as teachers and providing guidance<br>-Safe family environment<br>-Modeling of sobriety<br>-Expression of praise<br>-Parental affection and specialness of the child<br>-Parental transmission of cultural expectations and values<br>-Opportunity to be involved in the community<br>-Provision of safe places for children<br>-Limit setting on alcohol behavior<br>-Availability of extracurriculars<br>-Cultural connectedness<br>-Involvement in traditional activities<br>-Identification with American Indian culture<br>-Involvement and importance of traditional spirituality

Hirchak, Katherine; Amiri, Solmaz; Espinoza, Judith; Herron, Jalene; Hernandez-Vallant, Alexandra; Cloud, Violette; Venner, Kamilla	2021	Total: 42,098 (7,307 AI/AN) Location: New Mexico	Cross-sectional survey	New Mexico Youth Risk and Resilience Survey	Opioid use	-Social connection<br>- Engaging in clubs, sports teams, church or temple, or other group activity outside of home and school<br>- Having a friend that really cares<br>- Parent or some other adult who is interested in schoolwork<br>- Community support<br>- Having clear rules at school
Komro, Kelli A.; Livingston, Melvin D.; Garrett, Brady A.; Boyd, Misty L	2016	Total: 952 (422 Native) Location: Oklahoma	School-based survey	Survey created by researchers	Alcohol use	-Perceived accessibility<br>- Parental communication
Komro, Kelli A.; Livingston, Melvin D.; Wagenaar, Alexander C.; Kominsky, Terrence K.; Pettigrew, Dallas W.; Garrett, Brady A.; Boyd, Billy J.; Boyd, Misty L.; Livingston, Bethany J.; Lynne, Sarah D.; Molina, Mildred Maldonado; Merlo, Lisa J.; Tobler, Amy L.	2017	Total: 1,623 (746 Native youth) Location: Oklahoma	Skills enhancement program and intervention	Communities Mobilizing Change for Alcohol (CMCA), and CONNECT	Alcohol use	-Exposure to CMCA and CONNECT showed significant reduction in probability over time of 30-day alcohol use and heavy episodic drinking (knowledge)
Kulis, Stephen S.; Jager, Justin; Ayers, Stephanie L.; Lateef, Husain; Kiehne, Elizabeth	2016	Total: 62,817 (2,047 American Indian youth) Location: Arizona	Cross-sectional, state-wide, school-based study	Arizona Youth Survey (AYS)	Substance Use	-Less exposure to substance offers<br>- Less antisocial behavior<br>- Supportive peer networks<br>- Parental support for alcohol abstinence
Kulis, Stephen; Hodge, David R.; Ayers, Stephanie L.; Brown, Eddie F.; Marsiglia, Flavio F.	2012	123 American Indian youth Location: Southwest United States	Randomized Controlled Trial	Self-administered pretest questionnaires	Substance use	-Spirituality and religious involvement<br>- Frequent attendance at religious services<br>- Traditional spiritual beliefs
LeMaster, Pamela L.; Connell, Cathleen M.; Mitchell, Christina M.; Manson, Spero M.	2002	2,390 American Indian youth Location: Western United States	Survey	Voices of Indian Teens Project Survey	Tobacco use	-Academic orientation<br>- Connected to American Indian culture

Martinez, Marcos J.; Ayers, Stephanie L.; Kulis, Stephen; Brown, Eddie	2015	155 Native youth Location: Southwest United States	Longitudinal randomized controlled trial		Substance use	-Strong peer injunctive norms<br>- Grandparent injunctive norms<br>- Parent injunctive norms
Napoli, Maria; Marsiglia, Flavio Francisco; Kulis, Stephen	2003	Total: 4,630 (243 Native youth) Location: Southwest United States	Survey		Drug use	-Strong sense of belonging in school
Sittner, Kelley J.	2016	619 Native youth Location: Northern Midwest United States and Canada	Longitudinal study	Healing Pathways Project	Alcohol, marijuana, and cigarette use	-Caretaker monitoring
Spillane, Nichea S.; Weyandt, Lisa; Oster, Danielle; Treloar, Hayley	2017	3,498 American Indian youth Location: United States	Survey	American Drug and Alcohol Survey	Stimulant Use	-Parental monitoring
Thurman, P; Green, V A	1997	87 American Indian youth Location: Southwest United States	Survey		Inhalant use	-Participation in tribal activities<br>- Cognitive ability<br>- Cognitive egocentrism
Tingey, Lauren; Cwik, Mary F.; Rosenstock, Summer; Goklish, Novalene; Larzelere-Hinton, Francene; Lee, Angelita; Suttle, Rosemarie; Alchesay, Melanie; Massey, Kirk; Barlow, Allison	2016	136 Native youth Location: Arizona	Cross-sectional case-control study	Audio Computer Assisted Self-interview	Alcohol use	-Having social problem-solving skills<br>- Having traditional American Indian values and practices<br>- Having a strong ethnic identity<br>- Family closeness<br>- Residential stability
Whitesell, Nancy Rumbaugh; Asdigian, Nancy L.; Kaufman, Carol E.; Big Crow, Cecelia; Shangreau, Carly; Keane, Ellen M.; Mousseau, Alicia C.; Mitchell, Christina M.	2014	381 Native youth Location: Northern Plains	Longitudinal study and school-based surveys		Substance use	-Prosocial peers<br>- Strong relationships with parents
Whitesell, Nancy Rumbaugh; Kaufman, Carol E.; Keane, Ellen M.; Crow, Cecelia	2012	1,278 Native youth	Survey		Substance Use	-Positive parent influences

Big; Shangreau, Carly; Mitchell, Christina M.		Location: Northern Plains				
